# Efficiency of Non-Contrast-Enhanced Liver Imaging Sequences Added to Initial Rectal MRI in Rectal Cancer Patients

**DOI:** 10.1371/journal.pone.0137320

**Published:** 2015-09-08

**Authors:** Gene-hyuk Kwon, Kyung Ah Kim, Seong Su Hwang, Soo Youn Park, Hyun A. Kim, Sun Young Choi, Ji Woong Kim

**Affiliations:** 1 Department of Radiology, St. Vincent's Hospital, College of Medicine, The Catholic University of Korea, Seoul, Korea; 2 Department of Radiology and Medical Research Institute, School of Medicine, Ewha Womans University, Seoul, Korea; 3 Department of Chiropractic, Graduate School of Health promotion, Hanseo University, Seosan-Si, Korea; Northwestern University Feinberg School of Medicine, UNITED STATES

## Abstract

**Purpose:**

The purpose of this study was to estimate the value of addition of liver imaging to initial rectal magnetic resonance imaging (MRI) for detection of liver metastasis and evaluate imaging predictors of a high risk of liver metastasis on rectal MRI.

**Methods:**

We enrolled 144 patients who from October 2010 to May 2013 underwent rectal MRI with T2-weighted imaging (T2WI) and diffusion-weighted imaging (DWI) (b values = 50, 500, and 900 s/mm^2^) of the liver and abdominopelvic computed tomography (APCT) for the initial staging of rectal cancer. Two reviewers scored the possibility of liver metastasis on different sets of liver images (T2WI, DWI, and combined T2WI and DWI) and APCT and reached a conclusion by consensus for different analytic results. Imaging features from rectal MRI were also analyzed. The diagnostic performances of CT and an additional liver scan to detect liver metastasis were compared. Multivariate logistic regression to determine independent predictors of liver metastasis among rectal MRI features and tumor markers was performed. This retrospective study was approved by the Institutional Review Board, and the requirement for informed consent was waived.

**Results:**

All sets of liver images were more effective than APCT for detecting liver metastasis, and DWI was the most effective. Perivascular stranding and anal sphincter invasion were statistically significant for liver metastasis (p = 0.0077 and p = 0.0471), while extramural vascular invasion based on MRI (mrEMVI) was marginally significant (p = 0.0534).

**Conclusion:**

The addition of non-contrast-enhanced liver imaging, particularly DWI, to initial rectal MRI in rectal cancer patients could facilitate detection of liver metastasis without APCT. Perivascular stranding, anal sphincter invasion, and mrEMVI detected on rectal MRI were important imaging predictors of liver metastasis.

## Introduction

Rectal cancer accounts for about one-third of all colorectal cancers and is a common malignancy and the fourth leading cause of cancer death worldwide [1]. Treatment strategies for rectal cancer differ according to tumor stage. Therefore, precise evaluation of disease status is important for treatment strategy planning. A common protocol for preoperative radiologic evaluation includes rectal MRI and endoluminal ultrasound (US) for local disease extent and computed tomography (CT) of the chest and abdomen to check for metastasis [1]. Abdominopelvic CT (APCT) is typically obtained to search for metastasis in the abdominal organs or lymph nodes, and chest CT is performed to identify lung metastasis. While this suite of imaging procedures may be helpful for the accurate tumor staging of rectal cancer, it results in high examination fees and radiation exposure. An evaluation protocol that is as effective but involves fewer radiologic examinations would be beneficial for rectal cancer patients. Magnetic resonance imaging (MRI) is particularly useful for the preoperative evaluation of rectal cancer because the rectum, compared with other regions of the colon, is fixed within the pelvic cavity and is less affected by bowel movement than other parts of the colon, thus resulting in fewer artifacts on MRI. The excellent diagnostic accuracy of rectal MRI for local tumor staging has been demonstrated [1–4]. APCT is less effective for evaluating a circumferential resection margin (CRM) than rectal MRI, and also exposes the patient to radiation. A previous report found that it was uncommon to detect significant extrahepatic pathologies on APCT, and those that were detected did not influence management [5]. Therefore, if adding liver imaging to routine rectal MRI could allow for effective detection of liver metastasis, APCT could be removed from the protocol, and both the number of overall procedures and radiation exposure could be reduced. Additionally, by relying only on MRI, contrast materials are not required, simplifying the clinical procedures. The purpose of this study was to estimate the additional value of liver imaging alongside an initial rectal MRI to detect liver metastasis and evaluate imaging predictors of a high risk of liver metastasis on rectal MRI.

## Materials and Methods

This retrospective study was approved by the Institutional Review Board of St Vincent’s hospital, the Catholic University of Korea. The requirement for informed consent from patients was waived because the research presented no more than a minimal risk of harm to subjects and involved no procedures outside of the research context. We recruited 234 patients who underwent rectal MRI between October 2010 and May 2013. Patients were excluded from the study if they underwent rectal MRI for reasons other than an initial diagnosis and staging of rectal cancer. In a total of 90 excluded patients, rectal MRI was performed as a follow-up study after treatment for rectal cancer in 62 patients and for the evaluation of perianal fistula (n = 2), submucosal tumor arising from the rectum (n = 7), rectal endometriosis (n = 2), residual tumor after EMR (n = 3) and anal cancer (n = 14) in 28 patients. Finally, 144 patients (91 males and 53 females; mean age = 65.5 years; range, 38–99 years) who underwent rectal MRI and APCT for the initial diagnosis and staging of rectal cancer were enrolled in this study. Liver metastasis was diagnosed based on pathologic examination of liver sections or further imaging with PET-CT, contrast-enhanced liver MRI, and follow-up APCT. For tumor-marker assessment, the carcinoembryonic antigen (CEA) level was determined (normal CEA range: 0–5 ng/ml).

### Image acquisition

MRI examination was performed using a 3.0 T magnetic resonance system (Verio; Siemens, Munich, Germany) with six-channel body-array coils. MRI scans for liver imaging included axial T2-weighted half-Fourier acquisition single-shot turbo spin-echo (HASTE) images (repetition time (TR), 900 ms; echo time (TE), 90 ms; slice thickness, 6 mm; flip angle, 160°; matrix, 384 × 173; echo train length (ETL), 256) and diffusion-weighted images (DWI) (TR, 4465 ms; TE, 73 ms; slice thickness, 6 mm; flip angle, 90°; matrix, 256 × 192; ETL, 1) with three diffusion weightings (b values = 50, 500, and 900 s/mm^2^). An apparent diffusion coefficient (ADC) map was generated automatically. Contrast material was not used for liver MR imaging. HASTE imaging of the liver requires about 1.5 minutes, and DWI requires 5–6 minutes. For rectal imaging, sagittal T2-weighted images (T2WI) (TR, 4500 ms; TE, 111 ms; slice thickness, 3 mm; flip angle, 106°; matrix, 384 × 384; ETL, 24), coronal T2WI (TR, 6500 ms; TE, 88 ms; slice thickness, 3 mm; flip angle, 123°; matrix, 448 × 269; ETL, 11), axial T2WI (TR, 5000 ms; TE, 93 ms; slice thickness, 3 mm; flip angle, 138°; matrix, 512 × 358; ETL, 15), and pre-contrast axial T1-weighted spin echo images (TR, 723 ms; TE, 13 ms; slice thickness, 3 mm; flip angle, 130°; matrix, 512 × 307; ETL, 4) were obtained. Gadoterate meglumine (Dotarem; Guerbet, Villepinte, France) at a dose of 0.1 mM/kg was injected intravenously at a flow rate of 2.0 ml/s, followed by a 20 ml saline flush. After contrast injection, sagittal fat-suppressed T1-weighted turbo-spin echo (TSE) images (TR, 784 ms; TE, 9 ms; slice thickness, 3 mm; flip angle, 124°; matrix, 320 × 224; ETL, 3), axial fat-suppressed T1-weighted TSE images (TR, 711 ms; TE, 11 ms; slice thickness, 3 mm; flip angle, 140°; matrix, 384 × 230; ETL, 3), and coronal fat-suppressed T1-weighted TSE images (TR, 750 ms; TE, 9.4 ms; slice thickness, 3 mm; flip angle, 125°; matrix, 384 × 230; ETL, 4) were performed for rectal evaluation.

APCT was performed through the entire abdomen and pelvis using one of several types of multi-detector CT scanners (Lightspeed VCT (n = 37); GE Medical Systems, Waukesha WI, USA; SOMATOM Sensation 16 (n = 11), Siemens; SOMATOM Definition Flash (n = 96), Siemens (n = 96)). Patients received 2 ml/kg (total volume less than 150 ml) of nonionic contrast material, such as iopromide (Ultravist 300; Bayer, Leverkusen, Germany), iodixanol 320 (Visipaque; GE Healthcare, Little Chalfont, UK), or iohexol (Iobrix 300; Accuzen, South Korea) administered with a power injector (EnVision; Medrad, Warrendale, PA, USA). APCT examinations were performed in one phase (portal phase) in six patients, in two phases (pre-contrast and portal phases) in 133 patients, and in four phases (pre-contrast, arterial, portal and equilibrium phases) in 5 patients (slice thickness of CT images, 3mm). Portal-phase images were obtained with a delay of about 70 seconds after contrast material administration.

### Image analysis

Two radiologists with 7 and 19 years of experience in abdominal radiology, who were blinded to the clinical data, reviewed the rectal MRI images. They then discussed and reached a conclusion by consensus for different analysis results. The reviewers assessed rectal MRI and additional liver scans; after an interval of 4 weeks, they next analyzed the CT images. The reviewers analyzed various features on rectal MRI, including T stage, N stage, distance between the tumor and mesorectal fascia (MRF), circumferential resection margin (CRM) invasion, perirectal fat infiltration, focal nodular extension of the tumor, lateral pelvic LN, regional LN, anal sphincter invasion, extramural vascular invasion on MRI (mrEMVI), perivascular stranding, perivascular soft tissue, location of the tumor (above peritoneal reflection, below, above and below), distance from the anal verge to the lowest tumor margin, mucinous rectal cancer, peritoneal invasion, adjacent organ invasion, and ascites. Focal nodular extension of the tumor refers to a focal protruding portion of the tumor with a nodular shape, which has a length of more than half the thickness of the short axis of the tumor, along the direction perpendicular to the long axis of the main tumor, as viewed on an axial image. Perivascular stranding was defined as stranding around vessels without a tumor signal in the peritumoral vessel lumen. Perivascular soft tissue was defined as soft tissue with a tumor signal around the vessel but no tumor signal in the vessel. mrEMVI was defined as a tumor signal within the peritumoral vessel lumen or irregular vessel contour or nodular expansion of a vessel by a definite tumor signal [6]. Mucinous rectal cancer on MRI was defined as a tumor containing a mucin pool occupying at least 50% of the pool mass and that shows a characteristic high signal intensity on T2WI rectal MRI [7, 8]. Reviewers scored the possibility of liver metastasis on additional liver images [only T2WI, only DWI, and combined T2WI and DWI (T2WI + DWI)] using a four-point scale: 1 = definitely absent; 2 = probably absent; 3 = probably present; 4 = definitely present (Table 1). A score of 1 or 2 represents a negative decision for liver metastasis, and a score of 3 or 4 represents a positive decision. If there was a focal lesion in the liver, a region of interest (ROI) within the margin of the lesion was drawn on an ADC map. The ROI was drawn for the largest of the liver lesions with the same character, and an ROI was drawn for each lesion if a patient had focal liver lesions with different characters.

**Table 1:** Scores of the probability of liver metastasis on CT and MRI liver images (four-point scale).

Score	Probability of liver metastasis
1	definitely absent
2	probably absent
3	probably present
4	definitely present

On CT, portal phase images were analyzed by reviewers, and the presence of liver metastasis was evaluated using the four-point scale described above. Extrahepatic lesions in the upper abdomen that did not include a within-scan range of rectal MRI were also evaluated.

### Statistical assessment

Interobserver agreement for imaging analysis between two reviewers was estimated using kappa (k)-values. The strength of concordance according to k-values was interpreted as follows: k < 0.21, poor; k = 0.21–0.40, fair; k = 0.41–0.60, moderate; k = 0.61–0.80, good; and k > 0.80, excellent. The sensitivity, specificity, positive predictive value (PPV), and negative predictive value (NPV) were calculated to estimate the diagnostic performance of CT and an additional liver MRI to detect liver metastasis from rectal cancer. The diagnostic accuracy for the detection of liver metastasis was based on the area under the receiver operating characteristic curve (AUC). The diagnostic performance of CT compared with each additional liver scan (T2WI, DWI, and T2WI + DWI) was assessed using McNemar’s test. Univariate analyses of the relationships between radiologic imaging factors and tumor markers and liver metastasis were performed. Multivariate analysis of logistic regression was performed using factors found to be significant in univariate analyses. The differences in ADC values between liver metastasis and other benign liver lesions were assessed statistically using the Wilcoxon rank-sum test. P-values less than 0.05 were deemed to indicate statistical significance. Statistical analyses were performed using SAS software, version 9.3 (SAS Institute, Cary, NC, USA) and the DTComPair package for R version 3.1.1 (R Foundation for Statistical Computing, Vienna, Austria, www.r-project.org).

## Results

**Fig 1:**
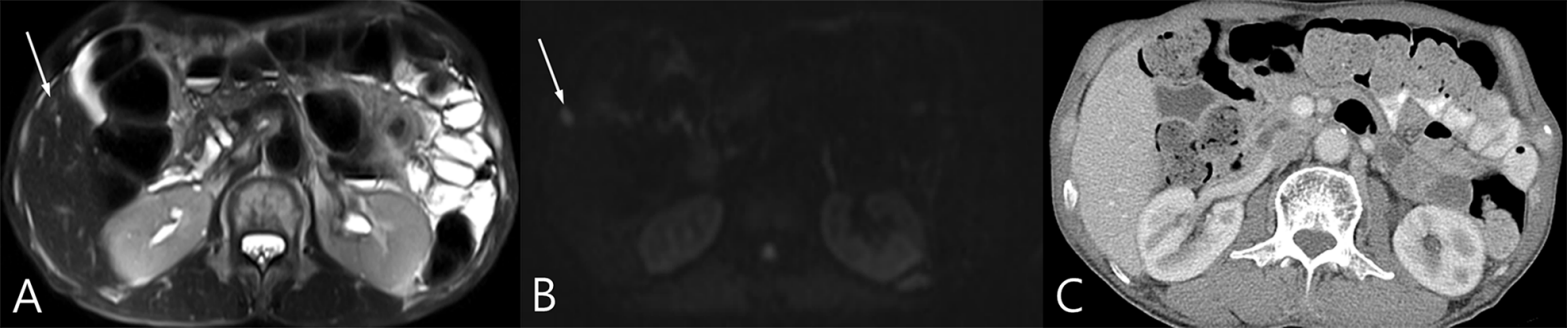
An 80-year-old male diagnosed with mid-rectal cancer with liver metastasis. (A) A 1 cm nodule with intermediate signal intensity in segment five of the liver is noted on T2WI. (B) The nodule shows high signal intensity with diffusion restriction on DWI (b = 900 s/mm^2^). (C) The nodule is suspected but not confirmed to be delineated on CT. The patient underwent lower anterior resection for rectal cancer and segmentectomy for a liver lesion in segment five of the liver after concurrent chemo-radiation therapy. The liver lesion was confirmed as liver metastasis on histologic evaluation.

Liver metastasis was diagnosed in 16 (14 males and 2 females, mean age = 63.9 years, range: 44–79) of 144 rectal cancer patients. These diagnoses were confirmed based on pathologic examination of liver resections in eight patients and additional imaging evaluation with PET-CT and enhanced liver MRI and the change-of-size of liver lesions on follow-up APCT in the other eight patients. Fifty patients had no focal liver lesion. Hepatic lesions, not including liver metastasis, that were detected on CT and additional liver scans included the following: hepatic cysts = 73, hemangioma = 5, and parenchymal calcification = 2. Interobserver agreement for the image analysis of rectal MRI involving additional liver scan was moderate to excellent (Table 2). The diagnostic performances of CT, T2WI, DWI, and T2WI + DWI to detect liver metastasis are presented in Table 3. The sensitivity, specificity, PPV, and NPV were 87.50, 97.66, 82.35, and 98.43, respectively, and the AUC for DWI was 0.93. DWI was superior to CT, T2WI, and T2WI + DWI in all parameters of diagnostic performance. Compared with the diagnostic performance between liver imaging sequences of MRI and CT, each measure of T2WI, DWI, and T2WI + DWI was more effective than CT for detecting liver metastasis (Fig 1); DWI was most effective (Table 4). The univariate analysis of rectal MRI findings and CEA levels found that CRM invasion (p = 0.0174), lateral pelvic LN (p = 0.0174), sphincter invasion (p = 0.0167), mrEMVI (p = 0.0014), perivascular stranding (p = 0.0013), perivascular soft tissue (p = 0.0136), and adjacent organ invasion (p = 0.0079) were statistically significant predictors of liver metastasis (Table 5). Multivariate logistic regression analysis of these seven significant factors found that perivascular stranding and sphincter invasion were the statistically significant predictors of liver metastasis (p = 0.0077, and p = 0.0471, respectively), with mrEMVI being marginally significant (p = 0.0534) (Table 5). The ADC value of liver metastasis (n = 16, mean ± standard deviation (SD), 1212.19 ± 786.45) was significantly lower than that of benign focal liver lesions (n = 80, mean ± SD, 2297.58 ± 653.65) (p < 0.0001). Extrahepatic lesions above the scan range of rectal MRI detected on APCT were mainly benign lesions of the following types: renal cysts = 56, gallbladder stones = 5, adenomyomatosis = 3, abdominal aneurysm = 2, biliary dilatation without obstructing lesion = 3, urinary stones = 4, adrenal hyperplasia = 1, duodenal diverticulum = 1, ischemic colitis = 1, benign pancreatic cystic lesion = 1, small bowel intussusception = 1, retroperitoneal varix = 1, and splenic cyst = 1. Significant extrahepatic lesions detected on APCT included ascending colon cancer (n = 1), cystic renal cell carcinoma (n = 1), periureteral metastasis (n = 1), and portocaval and retrocaval lymph node metastasis (n = 1).

**Table 2:** Interobserver agreement concerning imaging analyses of rectal MRI involving additional liver imaging.

Imaging findings	Kappa value (95% CI)
Liver lesion (T2WI)	0.87 (0.74–0.99)
Liver lesion (DWI)	0.97 (0.90–1.00)
Liver lesion (T2WI + DWI)	0.90 (0.78–1.00)
Liver lesion (CT)	0.90 (0.79–1.00)
T stage	0.92 (0.86–0.98)
N stage	0.88 (0.81–0.94)
CRM invasion	0.87 (0.78–0.97)
Perirectal fat infiltration	0.82 (0.70–0.94)
Nodular extension	0.68 (0.51–0.85)
Lateral pelvic LN	0.73 (0.59–0.88)
Regional LN	0.98 (0.94–1.00)
Sphincter invasion	0.93 (0.83–1.00)
Thrombi in vessel	0.68 (0.44–0.92)
Perivascular stranding	0.91 (0.84–0.98)
Perivascular soft tissue	0.95 (0.87–1.00)
Tumor location	0.94 (0.89–0.99)
Mucinous rectal cancer	0.96 (0.87–1.00)
Peritoneal invasion	0.92 (0.83–1.00)
Adjacent organ invasion	0.96 (0.89–1.00)
Ascites	0.92 (0.76–1.00)

CI, confidence interval.

**Table 3:** Diagnostic performance of T2WI, DWI, and CT for detecting liver metastasis.

Diagnostic performance	Sensitivity	Specificity	PPV	NPV	AUC
T2WI	81.25 (54.35–95.95)	86.09 (91.12–98.72)	72.22 (46.52–90.31)	97.62 (93.20–99.51)	0.89 (0.79–0.99)
DWI	87.50 (61.65–98.45)	97.66 (93.30–99.51)	82.35 (56.57–96.20)	98.43 (94.43–99.81)	0.93 (0.84–1.00)
T2WI + DWI	87.50 (61.65–98.45)	96.88 (92.19–99.14)	77.78 (52.36–93.59)	98.41 (94.38–99.81)	0.92 (0.84–1.00)
CT	43.75 (19.75–70.12)	91.41 (85.14–95.63)	38.89 (17.30–64.25)	92.86 (86.87–96.68)	0.68 (0.55–0.80)

Values in parentheses indicate the 95% confidence intervals.

PPV, positive predictive value;

NPV, negative predictive value;

AUC, area under the curve.

**Table 4:** Comparison of the diagnostic performance in terms of detecting liver metastasis among T2-weighted imaging, diffusion-weighted imaging, and CT.

Comparison of diagnostic performance	Sensitivity (p-value)	Specificity (p-value)	PPV (p-value)	NPV (p-value)	AUC (p-value)
T2WI vs. CT	0.0143	0.0833	0.0067	0.0103	0.0010
DWI vs. CT	0.0082	0.0325	0.0020	0.0057	0.0001
T2WI +DWI vs. CT	0.0082	0.0707	0.0054	0.0058	0.0002

PPV, positive predictive value;

NPV, negative predictive value;

AUC, area under the curve.

**Table 5:** Univariate and multivariate logistic regression of imaging findings on MRI and tumor markers for predicting liver metastasis.

Imaging findings and tumor marker	Univariate Analysis		Multivariate Analysis	
	Odds ratio (95% CI)	*p*-value	Odds ratio (95% CI)	*p*-value
T stage				
2	1			
3	4.07 (0.50–33.44)	0.1917		
4a	5.50 (0.46–66.32)	0.1796		
4b	13.20 (1.32–132.01)	0.0281		
N stage				
0	1			
1	Infinity	0.9428		
2	1.21 (0.40–3.68)	0.7341		
3	Infinity	0.9920		
Distance to MRF* (n = 104)	0.49 (0.15–1.57)	0.2265		
CRM invasion	3.75 (1.26–11.14)	0.0174*	0.97 (0.21–4.55)	0.9728
Perirectal fat infiltration	1.87 (0.40–8.74)	0.4255		
Nodular extension	3.18 (0.98–10.35)	0.0544		
Lateral pelvic LN	3.75 (1.26–11.14)	0.0174*	1.03 (0.26–4.17)	0.9622
Regional LN	1.33 (0.35–4.97)	0.6750		
Sphincter invasion	4.39 (1.31–14.78)	0.0167*	5.39 (1.02–28.39)	0.0471†
Thrombi in vessel	13.89 (2.78–69.46)	0.0014*	11.07 (0.97–127.06)	0.0534
Perivascular stranding	7.11 (2.15–23.44)	0.0013*	8.15 (1.74–38.15)	0.0077†
Perivascular soft tissue	4.20 (1.34–13.13)	0.0136*	0.29 (0.04–2.12)	0.2220
Tumor location				
below peritoneal reflection	1			
above and below	1.49 (0.46–4.84)	0.5043		
above reflection	0.54 (0.13–2.21)	0.3894		
Distance from AV	0.41 (0.14–1.26)	0.1201		
Mucinous rectal cancer	2.72 (0.66–11.17)	0.1642		
Peritoneal invasion	1.25 (0.33–4.77)	0.7480		
Adjacent organ invasion	5.36 (1.55–18.51)	0.0079*	2.65 (0.46–15.35)	0.278
Ascites	Infinity	0.9794		
CEA level^†^ (n = 134)	Infinity	0.9343		

CI, confidence interval;

*, statistically significant factor on univariate analysis;

^†^, statistically significant factor on multivariate analysis.

## Discussion

Distant metastasis from rectal cancer usually develops in the liver or lungs [9]. Chest CT and APCT are needed for preoperative evaluation, and the National Comprehensive Cancer Network (NCCN) guidelines recommend these examinations for rectal cancer evaluation. Although early cancer can be detected on endoscopic findings, the tumor may have spread beyond the extent expected based on the T stage [10]. Preoperative evaluation of the abdomen and chest is needed regardless of the T stage of primary rectal cancer. Small indeterminate hepatic lesions less than 1 cm in size in patients with colorectal cancer are frequently detected on preoperative APCT to evaluate liver metastasis [11]. However, most of these small indeterminate hepatic lesions are benign [11]. The main purpose of performing preoperative APCT in rectal cancer patients is to assess liver metastasis; however, CT may have limitations for the characterization of small hepatic lesions, an issue that needs further evaluation. Ultimately, the limitation of APCT results in higher examination fees and increased radiation exposure. Our results suggest the feasibility of addition of non-contrast-enhanced liver imaging to rectal MRI as an alternative method for preoperative APCT for patients with no focal liver lesion or benign liver lesions. Evaluations for liver metastasis that used DWI, T2WI, and T2WI + DWI produced results superior to those of APCT, particularly DWI, which showed the best diagnostic performance. This result suggests that a combination of DWI and T2WI actually decreases the confidence level for liver metastasis. The addition of liver imaging, such as DWI, T2WI, or even DWI + T2WI, to the initial diagnostic rectal MRI takes little extra time—less than 10 minutes—and does not require the use of contrast material. Therefore, these techniques are good alternatives to APCT for liver evaluation.

**Fig 2:**
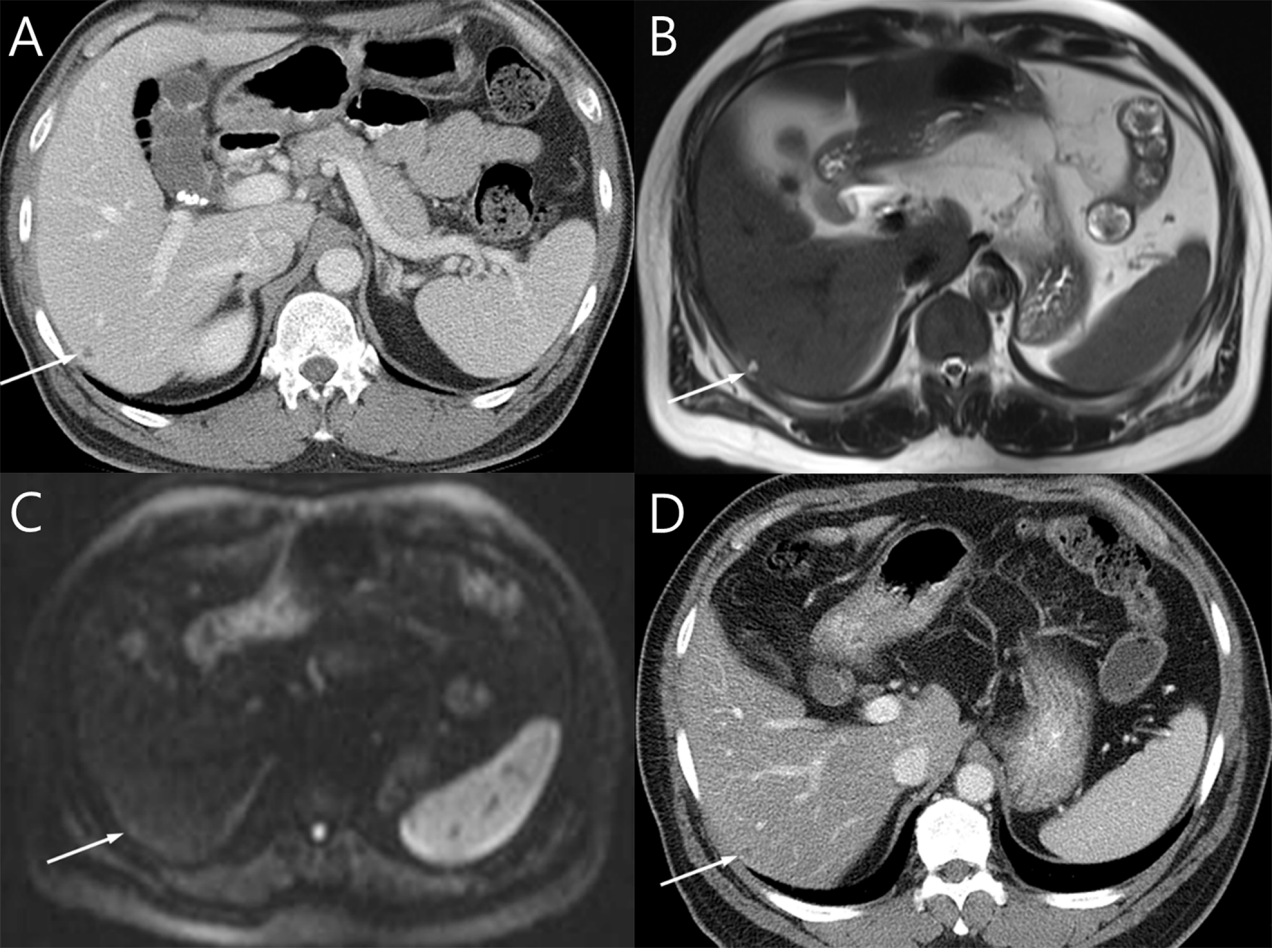
A 56-year-old male diagnosed with distal rectal cancer. (A) A 0.6 cm low attenuating nodule is noted in segment six of the liver on CT. It is difficult to determine whether it is a benign lesion or a small liver metastasis on CT. (B) The lesion shows high signal intensity on T2WI. (C) The nodule has high signal intensity on DWI (b = 50, 500 s/mm^2^) but iso-signal intensity on DWI (b = 900 s/mm^2^) without diffusion restriction. (D) The small nodule shows no interval change on follow-up CT obtained 2 years after initial CT.

**Fig 3:**
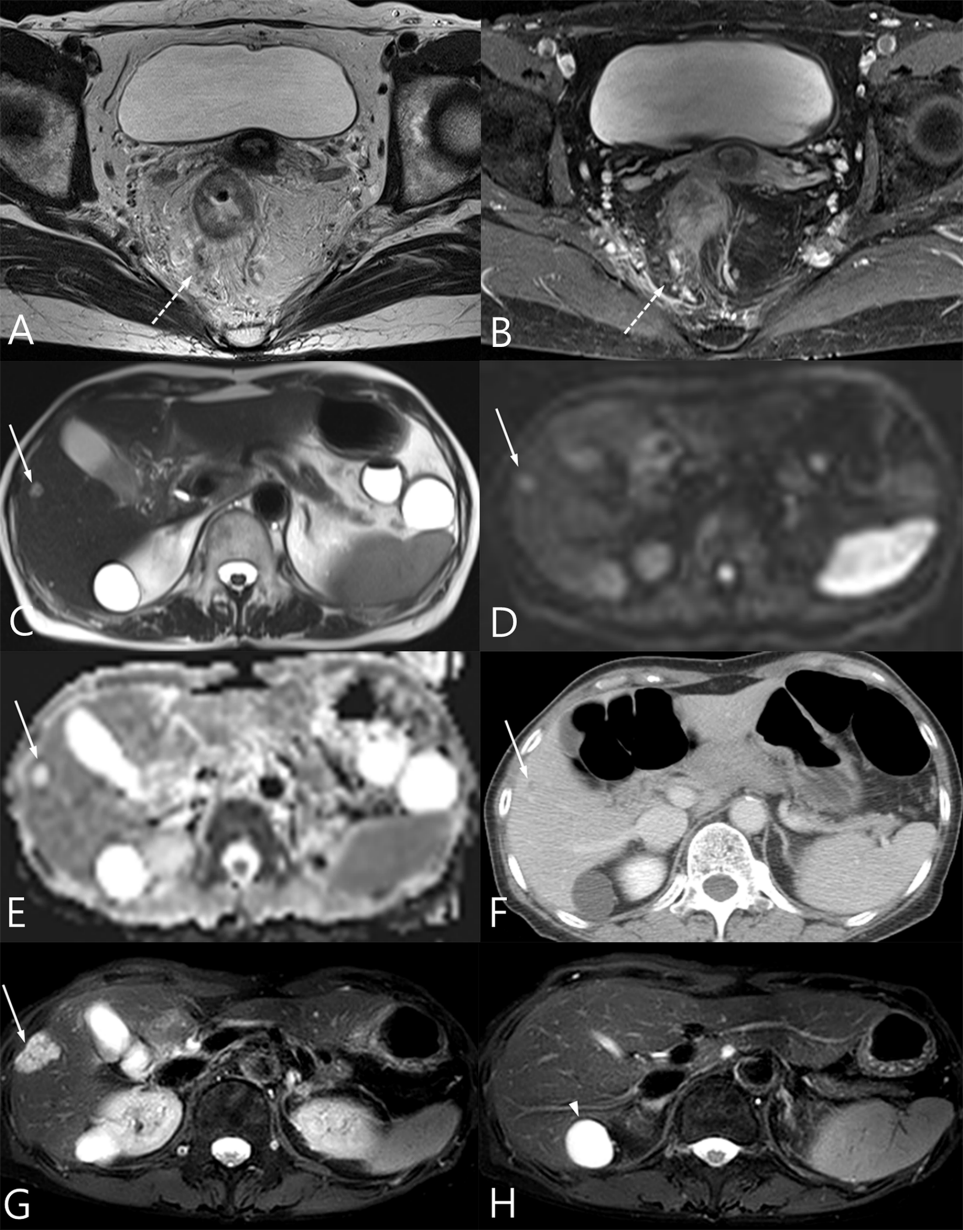
A 65-year-old female diagnosed with distal rectal cancer. (A) Perivascular stranding around the small vessels with a signal void on T2WI was noted. (B) Vessels around the rectum show contrast filling after contrast injection. (C) A small nodule with high signal intensity on T2WI is noted in segment five. (D-E) The lesion shows high signal intensity on DWI (b = 900 s/mm^2^) (D) without diffusion restriction on the ADC map (E). (F) The nodule shows focal peripheral enhancement in segment five on CT. The lesion was considered to be a hemangioma. (G) At the 2-year follow up, the lesion appeared to be enlarged on T2WI liver MRI after the patient underwent a laparoscopic abdominal transanal proctosigmoidectomy with coloanal anastomosis. The lesion was confirmed as liver metastasis of mucinous adenocarcinoma after liver segmentectomy. (H) Another lesion with high signal intensity on segment one on T2WI was also resected and diagnosed as a biliary cyst.

DWI is excellent for detecting lesions. A focal liver lesion can be considered benign if it shows high signal intensity at a low b value and substantial signal loss at a high b value. Malignant lesions show no or a minimal signal drop at a high b value [12]. Nevertheless, DWI has a limited ability to characterize such lesions [13]. However, a previous report revealed that unenhanced hepatic MRIs had a very negative predictive value for detection of liver metastasis at the preoperative evaluation stage [14]. This is similar to our results in terms of the high specificity and negative predictive value of DWI for predicting liver metastasis. The results indicate that unenhanced MRI is insufficient for accurate diagnosis of liver metastasis but facilitates identification of patients without liver metastasis as being negative for liver metastasis. The high negative predictive value of unenhanced MRI regarding the detection of liver metastasis may be due to the high prevalence of hepatic cysts relative to focal liver lesions among older patients. In this study, benign lesions were assumed to be mainly hepatic cysts, which were detected in 73 of 94 patients with focal liver lesions. DWI and T2WI can differentiate between numerous hepatic cysts with relatively high signal intensity (Figs 2 and 3). Liver metastasis of mucinous cancer showing high signal intensity on DWI and T2WI can resemble a hepatic cyst or hemangioma, but is rare (Fig 3). By successfully excluding hepatic cysts, these techniques may reduce the number of falsely identified rectal cancer patients selected for further evaluation for liver metastasis. However, if a focal liver lesion cannot be deemed benign confirmatively without suspicion, a suitable strategy for obtaining an accurate diagnosis is to perform further evaluations, such as liver MRI or CT with contrast enhancement.

Problematic extrahepatic lesions were rare among the analyzed rectal cancer patients. Most of the detected extrahepatic lesions did not affect treatment, even if detection was delayed. This result is similar to a previous report that a complete abdominal scan may be more effective for the selection of rectal cancer patients for liver resections if liver metastasis is isolated [5]. These data suggest the need to reduce the use of APCT in rectal cancer patients. Four significant extrahepatic lesions beyond the scan range of rectal MRI were found: an ascending colon cancer (n = 1), a cystic renal cell carcinoma (n = 1), a periureteral metastasis (n = 1), and a portocaval and retrocaval lymph node metastasis (n = 1). Ascending colon cancer was detected on colonoscopy, and other lesions were included within the additional liver scan. In the cases of periureteral metastasis and LN metastasis of the portocaval and retrocaval LNs, the primary cancer was advanced with pelvic organ invasion, suggesting the high probability of distant metastasis, which requires further evaluation of the upper abdomen and chest.

Rectal MRI predictors associated with a high risk of liver metastasis included perivascular stranding and sphincter invasion; mrEMVI was a marginally significant predictor. Perivascular stranding in this study was similar to the MRI extramural vascular invasion score of 2, as described by Smith et al. [6, 15]. This finding may be due to detection on MRI of extramural vascular invasion in a tiny vessel, in which it could be detected by microscopic examination [15]. The presence of perivascular stranding may be associated with microvascular tumor invasion prior to gross tumor thrombi formation within the vessel that can be visualized on MRI. Finally, perivascular stranding may be a high-risk factor for liver metastasis.

Sphincter invasion was also a significant predictor of liver metastasis in the present study. This result is somewhat unexpected. A previous report described T1 colorectal carcinomas with lymphovascular invasion and location in the lower third of the rectum as having a high risk for lymph node metastasis [16]. The rectum, particularly the distal-third portion, is drained by veins emptying into the inferior vena cava [9], which can facilitate liver metastasis. The significance of sphincter invasion in the present study may be due to the location of the tumor in the lower third of the rectum. However, the location of the tumor based on peritoneal reflection was not significantly predictive of liver metastasis. mrEMVI is known to be a poor prognostic factor for rectal cancer patients, and its presence increases the likelihood of development of metastasis [6, 17]. Tumor thrombi within the affected vessels on rectal MRI, even high-spatial-resolution MRI, were rarely detected and, in this study, were not easy to directly detect in peritumoral veins with a small diameter. Nevertheless, mrEMVI was marginally significant as a predictor of liver metastasis. These findings on rectal MRI suggest a high probability of liver metastasis and encourage careful evaluation for liver metastasis. Additionally, use of liver imaging plus rectal MRI may increase the confidence level of detection of liver metastasis from intermediate liver lesions.

As a limitation, the slice thickness of DWI and T2WI was 6 mm, which may have been too large to identify small focal liver lesions. It may be appropriate to reduce the slice thickness of liver images to avoid small metastases being missed, although in this case the slice thickness could not be changed due to the retrospective nature of this study.

In conclusion, the addition of non-contrast-enhanced liver imaging, particularly DWI, to initial rectal MRI in rectal cancer patients was effective for detecting liver metastasis by successfully excluding cases of benign hepatic lesions without the need for APCT. Perivascular stranding, anal sphincter invasion, and mrEMVI were important imaging predictors of liver metastasis from rectal MRI.
